# Supplementary material for: Ionic Dioxidovanadium(V) Complexes with Schiff-Base Ligands as Potential Insulin-Mimetic Agents—Substituent Effect on Structure and Stability
Source: Molecules. 2022 Oct 16;27(20):6942. doi: 10.3390/molecules27206942 (PMC9607307; doi:10.3390/molecules27206942)
Supplement: Supplementary file 1 [file molecules-27-06942-s001.zip › molecules-1965820-supplementary.pdf]

Supplementary Materials

Ionic Dioxidovanadium(V) Complexes with Schiff-Base Ligands as Potential Insulin-Mimetic Agents—Substituent Effect on Structure and Stability

Anna Jurowska [1,*], Janusz Szklarzewicz [1], Maciej Hodorowicz [1], Wiktoria Serafin [1], Ennio Zangrando [2] and Ghodrat Mahmoudi [3,*]

[1] Faculty of Chemistry, Jagiellonian University, Gronostajowa 2, 30-387 Kraków, Poland; janusz.szklarzewicz@uj.edu.pl (J.S.); hodorowm@chemia.uj.edu.pl (M.H.); wiktoria.serafin@student.uj.edu.pl (W.S.)

[2] Department of Chemical and Pharmaceutical Sciences, University of Trieste, Via L. Giorgieri 1, 34127 Trieste, Italy; ezangrando@units.it

[3] Department of Chemistry, Faculty of Science, University of Maragheh, Maragheh P.O. Box 55181-83111, Iran

* Correspondence: jurowska@chemia.uj.edu.pl (A.J.); ghodratmahmoudi@gmail.com or ghodratmahmoudi@maragheh.ac.ir (G.M.)

Table S1. Hydrogen bonds for complexes **1–4** [Å / °].

D-H...A	d(D-H)	d(H...A)	d(D...A)	<(DHA)
**1**				
N(3)-H(3n)...O(4)	0.88(2)	1.85(2)	2.7293(15)	176.2(19)
O(3)-H(3o)...N(2)	0.83(3)	1.83(3)	2.5857(15)	150(2)
C(4)-H(4)...O(4)#1	0.95	2.55	3.2405(16)	130.0
C(8)-H(8)...O(4)#1	0.95	2.45	3.1938(16)	134.6
C(14)-H(14b)...Cl(1)#1	0.99	2.90	3.7913(14)	150.6
C(18)-H(18a)...O(2)#2	0.99	2.49	3.4485(18)	164.1
C(18)-H(18b)...O(3)#3	0.99	2.62	3.4289(18)	138.8
C(20)-H(20c)...O(1)	0.98	2.50	3.4444(18)	161.4
#1 -x,-y,-z #2 x-1,y,z #3 x-1/2,-y+1/2,z+1/2				
**2**				
N(3)-H(3n)...O(4)	1.00	1.71	2.706(2)	178.6
O(3)-H(3o)...N(2)	0.87(4)	1.78(4)	2.572(2)	150(3)
C(7)-H(7)...O(5)#1	0.95	2.59	3.240(3)	125.5
C(26)-H(26a)...O(3)#2	0.99	2.50	3.297(3)	137.7
C(28)-H(28a)...O(3)#2	0.99	2.51	3.351(3)	142.3
C(18)-H(18)...Cl(1)#3	0.95	2.98	3.585(2)	123.1
C(31)-H(31a)...O(4)#4	0.98	2.55	3.408(3)	145.5
C(30)-H(30a)...O(5)#4	0.99	2.55	3.359(3)	139.0
C(30)-H(30b)...O(1)#4	0.99	2.66	3.185(2)	113.4
#1 -x+2,-y+1,-z+1 #2 -x+1,-y+1,-z+1 #3 x-1,y,z-1 #4 -x+1,-y,-z+1				
**3**				
N(3)-H(3n)...O(4)	0.81(2)	1.98(3)	2.787(2)	172(2)
O(3)-H(3o)...N(2)	0.83(3)	1.88(3)	2.6059(18)	146(3)
C(8)-H(8)...O(4)#1	0.95	2.60	3.309(2)	131.9
C(3)-H(3)...O(4)#1	0.95	2.49	3.238(2)	136.1
C(29)-H(29b)...O(6)#1	0.99	2.35	3.201(2)	143.4
C(31)-H(31a)...O(2)#2	0.99	2.54	3.497(2)	163.3
C(26)-H(26a)...O(7)#3	0.98	2.47	3.398(2)	158.3
C(30)-H(30b)...O(1)	0.98	2.57	3.536(2)	168.6
#1 -x+1,-y+1,-z+1 #2 x-1,y,z #3 x-1,y+1,z				
**4**				
N(3)-H(3n)...O(4)	0.91(3)	1.77(3)	2.686(2)	176(3)
O(3)-H(3o)...N(2)	0.78(4)	1.86(4)	2.567(2)	150(4)
C(2)-H(2)...O(5)#1	0.95	2.58	3.249(3)	127.6
C(26)-H(26a)...O(5)#2	0.99	2.53	3.380(3)	143.2

C(28)-H(28a)...O(3)#3	0.99	2.49	3.361(3)	146.6
C(17)-H(17)...Br(1)#4	0.95	3.07	3.760(2)	130.7
C(30)-H(30a)...Br(1)#5	0.99	3.14	3.910(2)	135.8
C(30)-H(30a)...O(3)#3	0.99	2.53	3.357(3)	140.8
C(30)-H(30b)...Cl(1)#6	0.99	2.94	3.846(2)	153.2
C(25)-H(25a)...O(4)#2	0.98	2.58	3.434(3)	144.9

#1 -x+2,-y+1,-z+1 #2 -x+1,-y,-z+1 #3 -x+1,-y+1,-z+1 #4 x-1,y,z-1 #5 x-1,y,z #6 x-1,y-1,z

Table S2. Results from Continuous Shape Measures calculation SHAPE v2.1.

PP-5	1 D5h	Pentagon
TBPY-5	3 D3h	Trigonal bipyramid
SPY-5	4 C4v	Square pyramid

Structure [ML5]	PP-5	TBPY-5	SPY-5
Complex **1**	29.043	4.128	1.309
Complex **2**	28.410	2.266	4.612
Complex **3**	28.777	4.357	1.264
Complex **4**	28.401	2.294	4.071

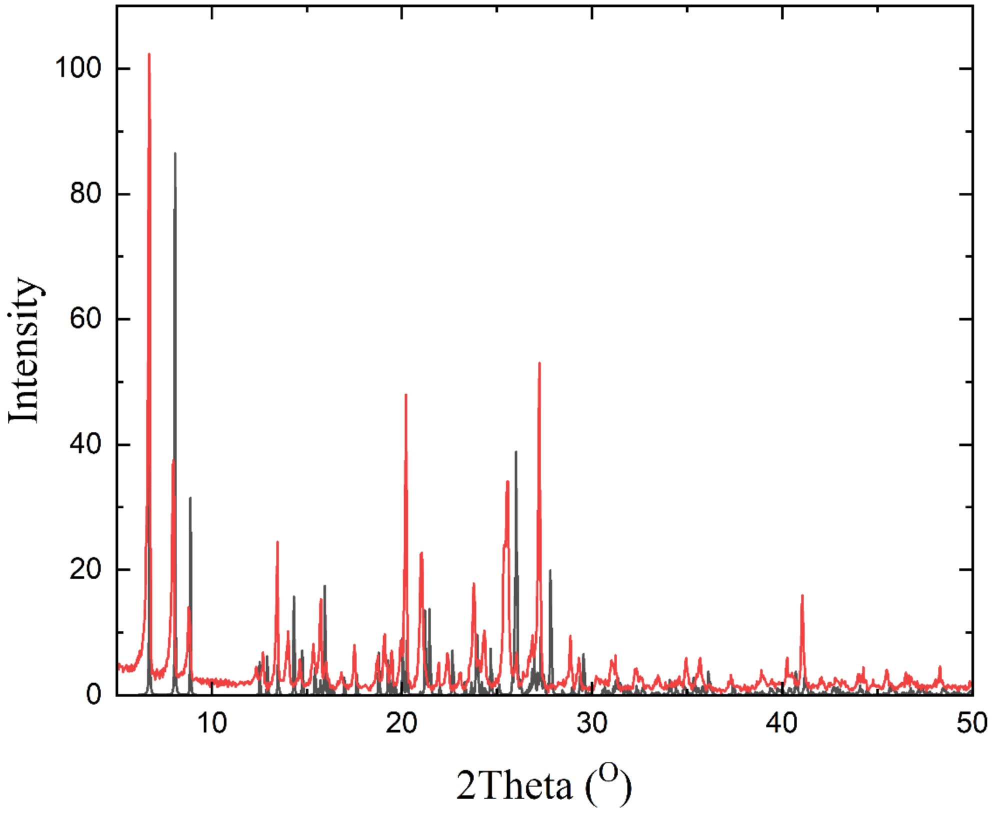

Figure S1. The comparison of powder diffractogram of **2** (red line) with powder diffractogram simulated from cif file for monocrystal of **2** (black line).

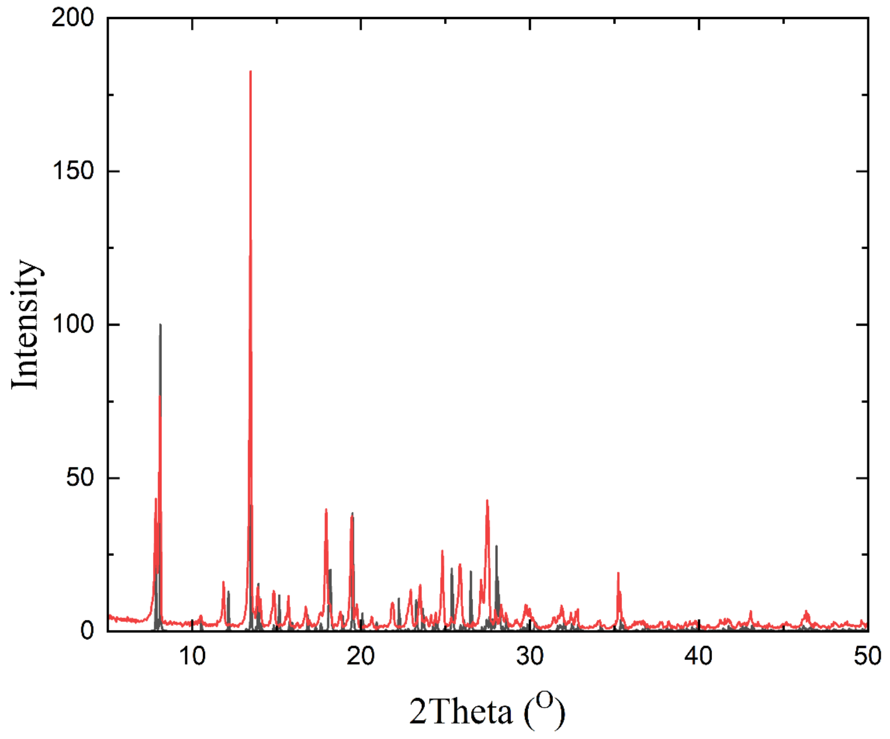

Figure S2. The comparison of powder diffractogram of **3** (red line) with powder diffractogram simulated from cif file for monocrystal of **3** (black line).

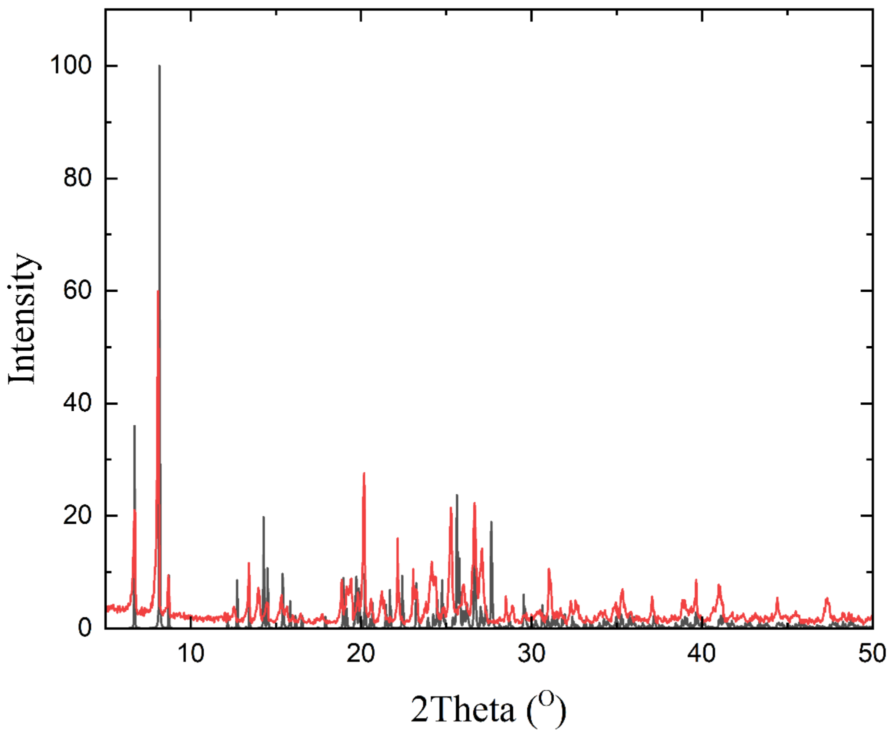

Figure S3. The comparison of powder diffractogram of **4** (red line) with powder diffractogram simulated from cif file for monocrystal of **4** (black line).
